# Prevalence of Sarcopenia Employing Population-Specific Cut-Points: Cross-Sectional Data from the Geelong Osteoporosis Study, Australia

**DOI:** 10.3390/jcm10020343

**Published:** 2021-01-18

**Authors:** Sophia X. Sui, Kara L. Holloway-Kew, Natalie K. Hyde, Lana J. Williams, Monica C. Tembo, Sarah Leach, Julie A. Pasco

**Affiliations:** 1 Deakin University, IMPACT—Institute for Mental and Physical Health and Clinical Translation, Geelong, VIC 3220, Australia; k.holloway@deakin.edu.au (K.L.H.-K.); natalie.hyde@deakin.edu.au (N.K.H.); l.williams@deakin.edu.au (L.J.W.); mctembo@deakin.edu.au (M.C.T.); julie.pasco@deakin.edu.au (J.A.P.); 2 GMHBA, Geelong, VIC 3220, Australia; SarahLeach@GMHBA.COM.AU; 3 Department of Medicine—Western Health, The University of Melbourne, St Albans, VIC 3021, Australia; 4 Department of Epidemiology and Preventive Medicine, Monash University, Prahran, VIC 3181, Australia; 5 University Hospital Geelong, Barwon Health, Geelong, VIC 3220, Australia

**Keywords:** sarcopenia, skeletal muscle, prevalence, muscle strength, physical functional performance, epidemiologic studies, aging

## Abstract

Background: Prevalence estimates for sarcopenia vary depending on the ascertainment criteria and thresholds applied. We aimed to estimate the prevalence of sarcopenia using two international definitions but employing Australian population-specific cut-points. Methods: Participants (*n* = 665; 323 women) aged 60–96 years old were from the Geelong Osteoporosis Study. Handgrip strength (HGS) was measured by dynamometers and appendicular lean mass (ALM) by whole-body dual-energy X-ray absorptiometry. Physical performance was assessed using gait speed (GS, men only) and/or the timed up-and-go (TUG) test. Using cut-points equivalent to two standard deviations (SDs) below the mean young reference range from the same population and recommendations from the European Working Group on Sarcopenia in Older People (EWGSOP), sarcopenia was identified by low ALM/height^2^ (<5.30 kg for women; <6.94 kg for men) + low HGS (<16 kg women; <31 kg men); low ALM/height^2^ + slow TUG (>9.3 s); low ALM/height^2^ + slow GS (<0.8 m/s). For the Foundation for the National Institutes of Health (FNIH) equivalent, sarcopenia was identified as low ALM/BMI (<0.512 m^2^ women, <0.827 m^2^ men) + low HGS (<16 kg women, <31 kg men). Receiver Operating Characteristic curves were also applied to determine optimal cut-points for ALM/BMI (<0.579 m^2^ women, <0.913 m^2^ men) that discriminated poor physical performance. Prevalence estimates were standardized to the Australian population and compared to estimates using international thresholds. Results: Using population-specific cut-points and low ALM/height^2^ + HGS, point-estimates for sarcopenia prevalence were 0.9% for women and 2.9% for men. Using ALM/height^2^ + TUG, prevalence was 2.5% for women and 4.1% for men, and using ALM/height^2^ + GS, sarcopenia was identified for 1.6% of men. Using ALM/BMI + HGS, prevalence estimates were 5.5–10.4% for women and 11.6–18.4% for men. Conclusions: This study highlights the range of prevalence estimates that result from employing different criteria for sarcopenia. While population-specific criteria could be pertinent for some populations, a consensus is needed to identify which deficits in skeletal muscle health are important for establishing an operational definition for sarcopenia.

## 1. Introduction

While sarcopenia is characterized by age-related declines in skeletal muscle mass, strength and function, currently, there is no unanimously agreed operational definition for sarcopenia [1,2,3,4]. Several operational definitions have been developed, notably by the European Working Group on Sarcopenia in Older People (EWGSOP1 and EWGSOP2) [3,5] and the Foundation for the National Institutes of Health (FNIH) [4]. Sarcopenia parameters usually include low muscle mass and low muscle strength or performance to identify sarcopenia, but different algorithms have been proposed. For example, the EWGSOP suggests that muscle mass be expressed relative to height, while the FNIH recommends adjustment by BMI. Such disparities contribute to poor agreement in the literature between prevalence estimates for sarcopenia [6,7,8]. Furthermore, the EWGSOP1, EWGSOP2 and FNIH present different cut-points for identifying low muscle mass, strength and/or performance, which have been identified on the basis of different criteria [4,5,9,10], using data largely from European or American populations [3,4,5]. However, in the more recent EWGSOP2, reference data have been drawn from a range of populations [5], including Australia [11]. We have recently published prevalence estimates for sarcopenia using criteria recommended by the EWGSOP1, EWGSOP2 and FNIH [8], but there remains a lack of consensus about whether or not population-specific reference data should be used to identify low muscle mass and function [9,12]. The Australian and New Zealand Society for Sarcopenia and Frailty Research (ANZSSFR) recently recommended EWGSOP1 criteria but suggested employing population-specific cut-points [12].

The aim of this study was to calculate and compare prevalence estimates of sarcopenia in a sample of older women and men using the EWGSOP and FNIH ascertainment criteria but employing cut-points derived from the same population.

## 2. Methods

### 2.1. Study Design

The Geelong Osteoporosis Study (GOS) is a population-based, prospective study in Australia. Further detailed information about the GOS is published elsewhere [13]. Participants were randomly selected from the electoral roll for the Barwon Statistical Division until there were at least 100 women and 100 men in each 5-year age group from 20 to 69 years and 200 of each sex for age groups 70–79 years and ≥80 years [13]. Inclusion criterion was a listing on the electoral roll for the Barwon Statistical Division; participants were excluded if residency in the region was less than 6 months or if they were not able to provide written informed consent. At baseline (1993–1997), an age-stratified sample of 1494 women was enrolled, with 77% response; in 2005, this sample was supplemented with a further 246 women aged 20–29 years. Baseline data for 1540 men were collected during 2001–2006 (67% response). Participants were followed-up every few years. The study was approved by the Barwon Health Human Research Ethics Committee. Written informed consent was obtained from all participants.

### 2.2. Participants

Cross-sectional data from the 15-year assessment waves for women and men were used in this analysis. To determine prevalence estimates of sarcopenia in older adults, we included data from the 15-year assessment for 323 women (ages 60–95 years), collected during 2010–2014, and for 342 men (ages 60–96 years), collected during 2016–2019. The sample was almost entirely Caucasian (~98%).

### 2.3. Measures

Weight and height were measured to the nearest ±0.1 kg and ±0.1 cm and body mass index (BMI) calculated as weight/height^2^ (kg/m^2^). Appendicular lean mass (ALM) (kg) was obtained from whole-body dual-energy X-ray absorptiometry (DXA; Lunar Prodigy-Pro, Madison, WI, USA), which provided lean mass measures for the arms and legs. Short-term precision (calculated as the coefficient of variation on repeated whole body scans) was 0.9% for ALM. ALM was expressed relative to height^2^ (ALM/height^2^, kg/m^2^) or relative to BMI (ALM/BMI, m^2^).

Handgrip strength (HGS) was measured using a hand-held analog dynamometer (Jamar, Sammons Preston, Bolingbrook, IL, USA) for women and a digital dynamometer (Vernier, LoggerPro3) for men. The testing procedure was demonstrated to participants before the measurement trials. With the participant seated in a comfortable position and the arm holding the dynamometer flexed at the elbow to 90 degrees, the participant was asked to squeeze the device as hard as possible for several seconds and the peak reading was recorded. This procedure was repeated for each hand. For women, the readings were performed in duplicate on each hand with no time interval between trials, and for men, trials were repeated in triplicate on each hand, holding the peak for 3 s with a 5-s interval between trials. The mean of the maximum value for each hand was used in further analyses. Measures from the Vernier device were transformed to Jamar equivalent values according to the following equation: HGS_Jamar_ (kg) = 9.50 + 0.818*HGS_Vernier_ (kg) + 8.80*Sex, where sex = 1 for men, which was developed by measuring the maximum HGS on each device for 45 adults aged 21–67 years [8].

The timed up-and-go (TUG) test was used as a measure of mobility but also includes static and dynamic balance [14]. This involved timing the participant (in seconds) to rise from a chair (without armrests), walk to a marked line (3 m distance), turn around, return to the chair and sit down. For men only, usual gait speed (GS, m/s) was also determined by measuring the time taken (in seconds) to walk a distance of 4 m. All measures were collected by trained personnel.

### 2.4. Population-Specific Cut-Points

Table 1 presents the Australian population-specific and international cut-points for the components of sarcopenia. Population-specific cut-points were determined as equivalent to 2 standard deviations (SDs) below sex-specific mean values for young reference groups (age ≤ 49 years) generated from the same population, as previously described [11,15,16,17]. For women, the cut-point for low HGS was <16 kg [16]. Using the same approach, the mean ±SD for HGS among 111 men (ages 33–49 years) was 44.8 ± 6.9 kg, and thus, the cut-point for low HGS was <31 kg. Low lean mass was identified as ALM/height^2^ <5.30 kg/m^2^ for women and 6.94 kg/m^2^ for men [11], and low ALM/BMI as <0.512 m^2^ for women and 0.827 m^2^ for men [15], corresponding to T-scores < −2 [11,15].

We used a cut-point of <0.8 m/s for GS to identify slowness (poor muscle performance) in line with extant literature [2,6,18]. The mean ±SD for TUG among women was 6.98 ± 1.14 s, and thus, slow TUG was identified as >9.3 s. We also used TUG as a proxy for GS [2,14] for men, and since the cut-points for slow GS are the same for both sexes in the literature [3,4,5], we used the same threshold for TUG for both women and men.

**Figure A1:**
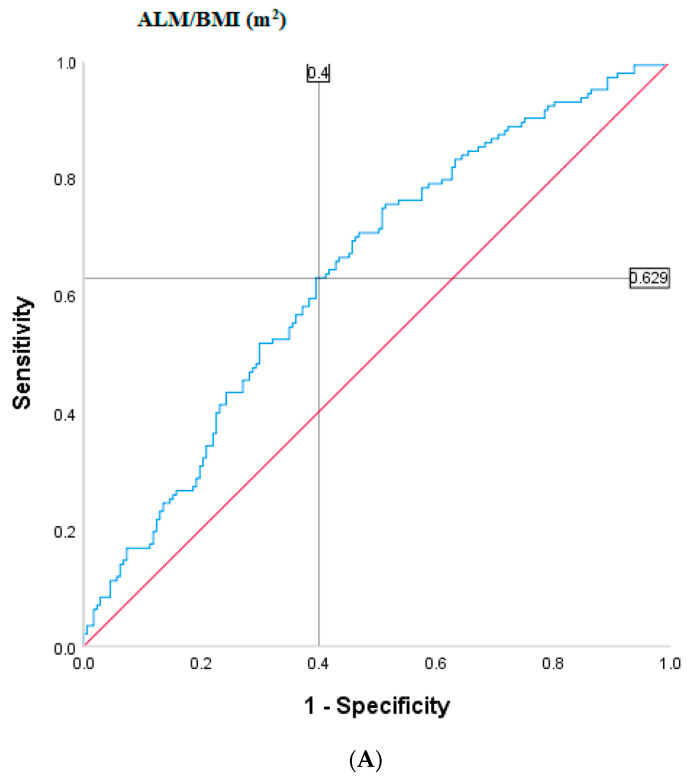

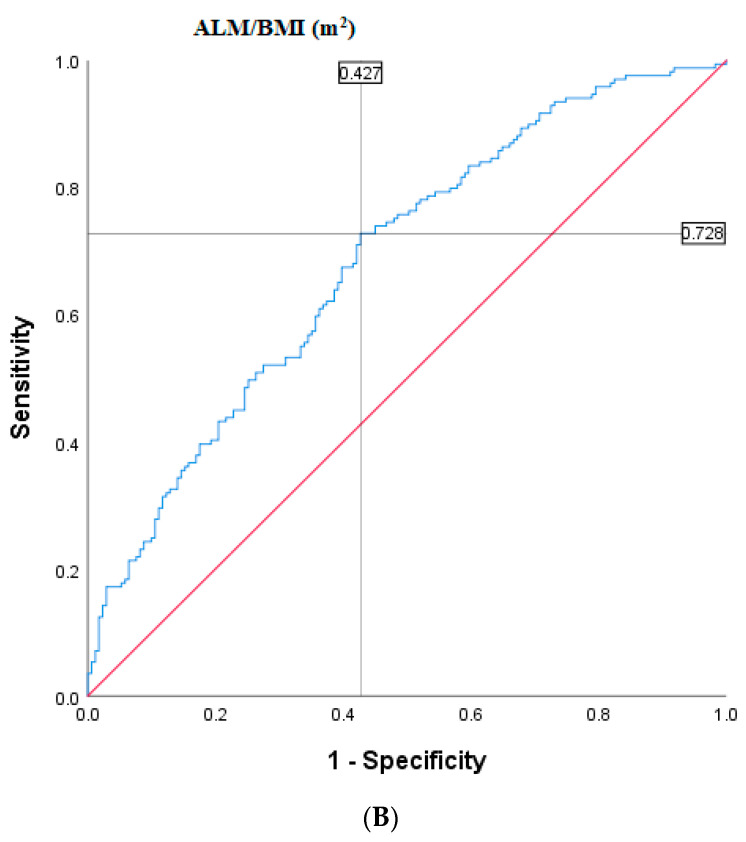
ROC analysis for optimal discrimination of slow timed up-and-go (TUG; slow TUG < 9.3 s) for ALM/BMI ((**A**,**B**) for women and men, respectively). (**A**) ALM/BMI that best predicted slow TUG (Women); (**B**) ALM/BMI that best predicted slow TUG (Men).

Furthermore, as the FNIH cut-points for ALM/BMI were identified on the basis of discriminating clinically significant weakness [18], we estimated cut-points for low ALM/BMI that best discriminated the presence or absence of slow TUG (>9.3 s) [2,6,9,18]. The locations of optimal cut-points were determined by the principle that the sensitivity and specificity are closest to the value of the area under the receiver operating characteristic (ROC) curve, and the absolute value of the difference between the sensitivity and specificity is the smallest [19]. The ALM/BMI that best predicted slow TUG was <0.579 m^2^ (sensitivity 0.63, specificity 0.60) for women and <0.913 m^2^ (sensitivity 0.73, specificity 0.57) for men (Appendix A Figure A1). The area under the ROC curve was 0.64 (95% CI 0.58–0.70) for women and 0.68 (0.63–0.74) for men (*p* < 0.001).

### 2.5. Sarcopenia Ascertainment

Based on EWGSOP1 [3] and EWGSOP2 [5], sarcopenia corresponds to low ALM/height^2^ and low HGS (ALM/height^2^ + HGS); low ALM/height^2^ and slow GS (ALM/height^2^ + GS); or low ALM/height^2^ and slow TUG (ALM/height^2^ + TUG). According to FNIH [4], sarcopenia is defined as low ALM/BMI and low HGS (ALM/BMI + HGS) (Table 1). Furthermore, severe sarcopenia was determined using a combination involving low lean mass, muscle strength and physical performance, that is, ALM/height^2^ + HGS + TUG for EWGSOP and ALM/BMI + HGS + TUG for FNIH.

### 2.6. Statistical Analysis

Data for women and men were analyzed separately. Histograms were used to check the distribution of data for normality. Means and SDs were presented for normally distributed data, and medians and interquartile ranges for skewed data. Prevalence for each age decade was calculated. Age-standardized prevalence estimates (mean and 95% confidence interval (CI)) were calculated according to 2011 census data from the Australian Bureau of Statistics [20]. Age-adjusted multivariable logistic regression models were developed to examine sex differences (pooled data) in the likelihood for sarcopenia. To compare prevalence estimates obtained with different cut-points, the kappa coefficient (κ) and 95% CIs were calculated and the strength of agreement was interpreted as small (κ < 0.40), medium (κ = 0.40–0.75) or high (κ > 0.75) [7]. Analyses were performed using SPSS (v24, IBM SPSS Statistics Inc., Chicago, IL, USA) and Minitab (v18, Minitab, State College, PA, USA).

## 3. Results

### 3.1. Participant Characteristics

Table 2 shows the participant characteristics. There were 12 women (3.7%) and 23 men (6.7%) with low ALM/height^2^, 70 women (21.7%) and 110 men (32.2%) with low ALM/BMI and 50 women (15.5%) and 87 men (25.6%) with low HGS. A slow TUG was recorded for 143 women (44.7%) and 169 men (49.7%) and a slow GS for 102 men (30.4%). Using the cut-point values obtained from ROC curves, 162 (50.2%) women and 197 (57.6%) men were identified as having low ALM/BMI_ROC_.

There was a pattern of increasing prevalence of sarcopenia with advancing age in both sexes across all the definitions (Table 3). The point estimates for men were higher than for women, especially for those aged ≥80 yr; however, 95% CIs for different age groups overlapped.

### 3.2. Sarcopenia Prevalence in Men Compared with Women

After adjusting for age, and according to FNIH-related definitions, men were more likely than women to have sarcopenia; for ALM/BMI + HGS, odds ratio (OR) 2.45 (95%CI 1.32–4.56; *p* = 0.005) and for ALM/BMI_ROC_ + HGS, OR 2.27 (95%CI 1.39–3.72; *p* = 0.001). When EWGSOP-related definitions were used, men appeared to be more likely than women to have sarcopenia, but differences were not significant; for ALM/height^2^ + HGS, OR 2.8 (95%CI 0.77–10.5; *p* = 0.11), and for ALM/height^2^ + TUG, OR 1.5 (95%CI 0.6–3.7; *p* = 0.37).

### 3.3. Age-Standardized Estimates of Sarcopenia

**Figure 1:**
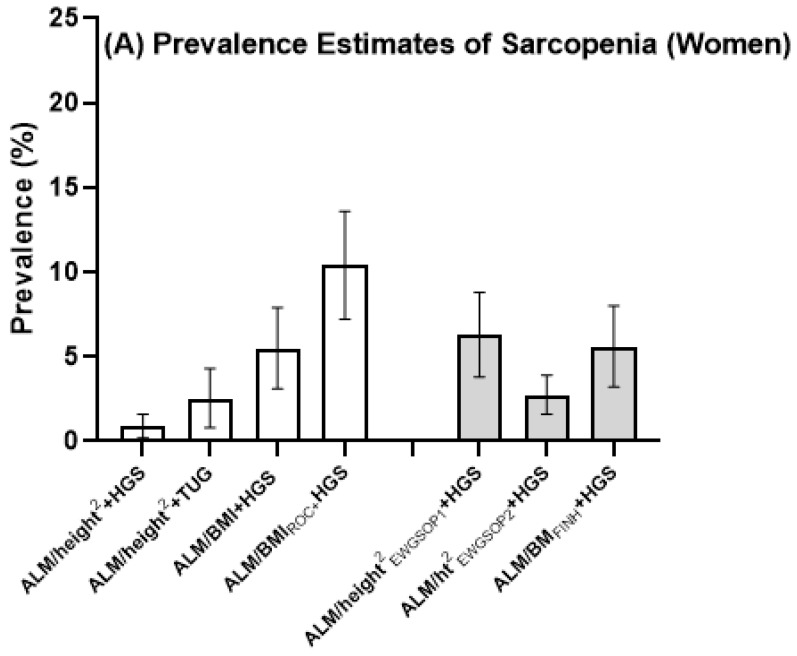

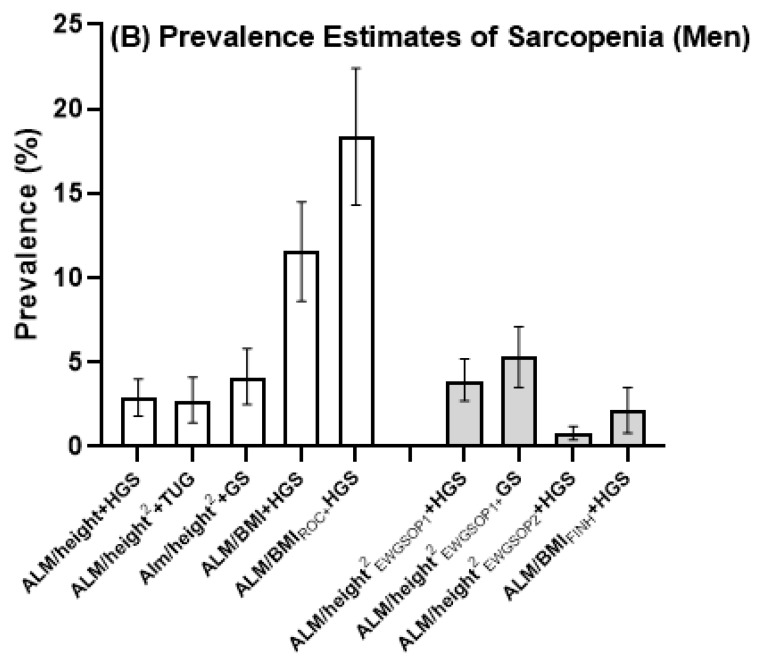
Prevalence estimates of sarcopenia for (**A**) women and (**B**) men aged 60 years and older. Error bars show 95% confidence intervals. Bars for estimates using population-specific cut-points are unshaded and those using international cut-points are shaded. EWGSOP_:_ European Working Group on Sarcopenia in Older People; FNIH: Foundation for the National Institutes of Health; ALM: appendicular lean mass; GS: gait speed; HGS: handgrip strength; BMI: body mass index; TUG: timed up-and-go.

The age-standardized estimates of sarcopenia according to different definitions and population-specific cut-points are shown in Table 3 and Figure 1. Using ALM/height^2^ + low HGS, point estimates for sarcopenia prevalence were 0.9% for women and 2.9% for men. Using ALM/height^2^ + TUG, estimates were 2.5% for women and 4.1% for men, and using ALM/height^2^ + GS, the estimate for men was 1.6%. Using ALM/BMI + HGS, point estimates ranged from 5.5% to 10.4% for women and from 11.6% to 18.4% for men. The prevalence estimates based on population-specific cut-points are shown in Figure 1 together with estimates based on recommended international criteria. Prevalence estimates using international cut-points (shown in Figure 1) have been published elsewhere [8].

### 3.4. Agreement

Table 4 shows the levels of agreement between different definitions of sarcopenia using international and population-specific cut-points. Levels of agreement ranged from poor through to high (κ = 0.1–1 for women and 0–0.8 for men). Note that the 100% agreement for women using the FNIH definition occurred because the international and population-specific thresholds were the same, even though they were obtained using different methods.

## 4. Discussion

We have reported sarcopenia prevalence in an Australian population using several cut-points for EWGSOP and FNIH definitions. Using these cut-points, we obtained substantial differences in prevalence estimates for sarcopenia, and the level of agreement between definitions varied widely. Using population-specific cut-points equivalent to T-scores <−2, the FNIH definition produced the greatest prevalence, while EWGSOP provided the lowest. As the cut-point for low ALM/BMI_ROC_ that discriminated slow TUG was greater than ALM/BMI T-score <−2, the prevalence estimates for sarcopenia were correspondingly higher, and this was mainly a consequence of low ALM/BMI_ROC_ among the elderly. Regardless, there was a pattern of increasing sarcopenia prevalence with advancing age across all definitions.

The higher prevalence estimates for sarcopenia for older ages was also found in a study in the Netherlands, where diagnostic criteria for sarcopenia influenced prevalence estimates in a middle-aged cohort (mean age 61.8 years for *n* = 329 women and 64.5 years for *n* = 325 men). The authors reported the prevalence of sarcopenia ranged from 0% to 15.6%, 0% to 21.8% and 0% to 25.8% in women aged <60, 60–69 and ≥70 years, respectively, and from 0% to 20.8%, 0% to 31.2% and 0% to 45.2% in men aged <60, 60–69 and ≥70 years, respectively. These results indicate an age-related increase in sarcopenia for all definitions reflecting a decline in muscle mass and performance with age [6,21,22,23].

For both women and men, when applying population-specific cut-points, we observed that for each age-decade, prevalence estimates were lower for EWGSOP than FNIH. The age-standardized estimates were lower according to EWGSOP than FNIH for both women and men. Dam et al. (2014) [7] of the FNIH research group reported that 2.3% of women and 1.3% of men (proportions outside the 95% CIs of our estimates) in their pooled samples from the USA were classified as having sarcopenia using FNIH, while the prevalence was 13.3% for women and 5.3% for men using EWGSOP1 (point estimates outside our 95% CIs for women, but not for men). Similarly, an Australian study by Sim et al. [2] found that FNIH diagnosed fewer women with sarcopenia than EWGSOP (9.4% vs. 24.1%). In addition, Sim et al. [2] applied Australian female population-specific definitions for FNIH (defined as ALM/BMI < 0.517 m^2^ + HGS < 17 kg) and EWGSOP (defined as ALM/height^2^ < 5.28 kg/m^2^ + HGS < 17 kg). However, the percentage was similar after harmonizing the cut-points.

Our results showed that overall, the agreement between FNIH and EWGSOP was poor, regardless of the cut-points employed. The poor agreement between the original EWGSOP (EWGSOP1) and FNIH definitions is well documented in a number of studies [6,7,8,24]. For example, Dam et al. [7] examined the difference between FNIH definitions and EWGSOP1. The agreement between the FNIH criteria (low HGS and low lean mass) and EWGSOP was poor in women (κ = 0.14) and medium in men (κ = 0.53). However, to our knowledge, this is the first study to examine the agreement between the EWGSOP and FNIH definitions after applying population-specific cut-points in an Australian setting. Masanes et al. [25] found that small differences in cut-points for low lean mass produced substantial variations in prevalence estimates for sarcopenia, and our findings are consistent with their results.

Although the cut-points recommended by EWGSOP [3,5] were adopted from different studies, the method for identifying deficits differed; while some identified low muscle mass and poor performance using the lower portion of the population distribution, the FNIH used a Classification and Regression Tree analysis [10] to identify clinically relevant criteria [4]. In our study, the population-specific cut-points were consistently identified using the lower portion of the population distribution, with the exception of ALM/BMI, where we also used ROC curves to identify low ALM/BMI values that corresponded to poor physical performance. There is still a need to reach a consensus as to which deficits in skeletal muscle health, and the extent of these deficits, are important in defining sarcopenia. Our results highlight disparities in prevalence estimates arising from the thresholds employed, suggesting that population-specific cut-points might be useful in certain populations.

Our study has both strengths and weaknesses. The participants were selected at random from the electoral roll and represent a broad adulthood age range. Almost the entire sample was Caucasian, and this might limit the generalization of our results to other ethnic groups in Australia and beyond. Whereas in this study, we used the mean of the maximum HGS for each hand as being indicative of strength, in some other studies, the maximum irrespective of handedness has been used. In recognition that the methods reported in the literature to identify maximum HGS vary, our choice of one method over another is a potential limitation. Prevalence data for sarcopenia in this study may have been influenced by differential participation and retention bias related to muscle health. Data were also lacking for participants who had physical impairments that prevented them from performance testing. Although data for women and men were pooled to identify sex differences, prevalence estimates for women and men have otherwise been analyzed separately as they were collected at different times.

In conclusion, this study takes a step towards a response to ANZSSFR’s call to investigate evidence-based cut-points for EWGSOP criteria for the populations of Australian and New Zealand [12]. We have provided population-based data which will help clinicians and researchers in the field establish new operational definitions for identifying individuals with sarcopenia in the Australian population. However, it is yet to be decided which deficits in skeletal muscle health are important in identifying sarcopenia. Until a universally agreed operational definition of sarcopenia exists internationally and in Australia, prevalence data should be reported with consideration of the ascertainment criteria used and, thus, interpreted in context.

## Figures and Tables

**Table 1:** Applied threshold values for women and men used in different definitions.

Population-Specific Cut-Points		
	Women	Men
ALM/height^2^ + HGS	<5.30 kg/m^2^ + <16 kg	<6.94 kg/m^2^ + <31 kg
ALM/height^2^ + TUG	<5.30 kg/m^2^ + >9.3 s	<6.94 kg/m^2^ + >9.3 s
ALM/height^2^ + GS	-	<6.94 kg/m^2^ + <0.8 m/s
ALM/BMI + HGS	<0.512 m^2^ + <16 kg	<0.827 m^2^ + <31 kg
ALM/BMI_ROC_ + HGS	<0.579 m^2^ + <16 kg	0.913 m^2^ + <31 kg
**International Cut-Points**		
ALM/height2EWGSOP1 + HGS (3)	<5.67 kg/m^2^ + <20 kg	<7.23 kg/m^2^ + <30 kg
ALM/height2EWGSOP1 + GS (3)	-	<7.23 kg/m^2^ + <0.8 m/s
ALM/height2EWGSOP2 + HGS (5)	<5.5 kg/m^2^ + <16 kg	<7.0 kg/m^2^ + <27 kg
ALM/height2EWGSOP2 + TUG (5)	<5.5 kg/m^2^ + >20 s	<7.0 kg/m^2^ + >20 s
ALM/BMIFNIH + HGS (4)	<0.512 m^2^ + <16 kg	<0.789 m^2^ + <26 kg

ALM: appendicular lean mass; ALM/height^2^: appendicular lean mass/height^2^; ALM/BMI: appendicular lean mass/body mass index; HGS: handgrip strength; TUG: timed up-and-go; GS: gait speed; EWGSOP_:_ European Working Group on Sarcopenia in Older People; FNIH: Foundation for the National Institutes of Health; ROC: receiver operating characteristic.

**Table 2:** Participant characteristics. Data are presented as mean (±SD) or median (IQR).

	Women (*n* = 323)	Men (*n* = 342)
Age (yr)	70 (64–75)	70 (66–78)
Weight (kg)	74.0 (±15.4)	83.9 (±13.8)
Height (m)	1.59 (±0.06)	1.73 (±0.07)
BMI (kg/m^2^)	29.0 (±5.8)	28.0 (±4.1)
HGS (kg)	21 (±6)	36 (±6)
ALM/height^2^ (kg/m^2^)	6.60 (±0.79)	8.25 (±0.93)
ALM/BMI (m^2^)	0.593 (±0.102)	0.888 (±0.124)
TUG (s)	9.1 (7.9–10.8)	9.2 (8.0–10.7)
Gait speed (m/s)	-	0.9 (±0.2)

BMI: body mass index; ALM: appendicular lean mass; HGS: handgrip strength; ALM/height^2^: appendicular lean mass/height^2^; ALM/BMI: appendicular lean mass/body mass index; TUG: timed up-and-go. Missing data: HGS *n* = 1 man; TUG *n* = 3 women, 2 men; GS *n* = 323 women, 7 men.

**Table 3:** Age- and sex-specific prevalence estimates of sarcopenia according to different assessment criteria.

Criteria	60–69 yr		70–79 yr		≥80 yr		All	Standardized Prevalence
Women	*n* = 151		*n* = 124		*n* = 48		*n* = 323	
	*n*, %,	95%CI	*n*, %,	95%CI	*n*, %,	95%CI	*n*, %,	Mean (%, 95%CI)
ALM/height^2^ + HGS	0 (0)	-	2 (1.6)	-	1 (2.1)	-	3 (0.9)	0.9 (0.2–1.6)
ALM/height^2^ + TUG	3 (2.0)	0.6–5.6	3(2.4)	0.7–6.7	2 (4.3)	0.9–13.9	8 (2.7)	2.5 (0.8–4.3)
ALM/BMI + HGS	3 (2.0)	0.6–5.5	5 (4.0)	1.6–8.9	8 (16.7)	8.2–29.3	16 (5.0)	5.5 (3.1–7.9)
ALM/BMI_ROC_ + HGS	5 (3.3)	1.3–7.7	8 (6.5)	3.3–12.4	16 (33.3)	21.5–47.7	29 (9.0)	10.4 (7.2–13.6)
Severe sarcopenia								
ALM/height^2^ + HGS + TUG	1 (0.8)	-	1 (2.1)	-	1 (2.1)	-	2 (0.6)	1.0 (0–2.1)
ALM/BMI + HGS + TUG	2 (1.3)	0.3–5.2	3 (2.4)	0.7–7.2	8 (17.0)	8.7–30.5	13 (4.1)	4.8 (2.6–6.9)
ALM/BMI_ROC_ + HGS + TUG	4 (2.7)	1.0–6.9	5 (4.0)	1.7–9.3	15 (31.9)	20.2–46.4	24 (7.5)	8.9 (6.0–11.8)
Men	*n* = 152		*n* = 117		*n* = 73		*n* = 342	
ALM/height^2^ + HGS	0 (0)	-	3 (2.6)	-	8 (11.1)	-	11 (3.2)	2.9 (1.8–4.0)
ALM/height^2^ + TUG	2 (1.3)	0.3–4.6	1 (0.9)	0.09–4.8	12 (16.4)	9.3–26.3	15 (4.4)	4.1 (2.4–5.8)
ALM/height^2^ + GS	0	-	0	-	6 (8.3)	-	6 (1.8)	1.6 (1.0–2.2)
ALM/BMI + HGS	4 (2.6)	0.9–6.4	13 (11.2)	6.4–18.0	26 (36.1)	25.7–47.6	43 (12.6)	11.6 (8.6–14.5)
ALM/BMI_ROC_ + HGS	13 (8.6)	5.0–14.2	21 (18.1)	12.1–26.1	33 (45.8)	34.7–57.4	67 (19.7)	18.4 (14.3–22.4)
Severe sarcopenia								
ALM/height^2^ + HGS + TUG	0	-	0	-	7 (9.7%)	-	7 (2.1)	1.9 (1.3–2.5)
ALM/BMI + HGS + TUG	2 (1.3)	0.3–5.2	9 (7.8)	4.1–14.2	25 (34.7)	24.7–46.4	36 (10.7)	9.5 (7.0–12.0)
ALM/BMI_ROC_ + HGS + TUG	4 (2.7)	1.0–6.9	14 (12.1)	7.3–19.4	32 (44.4)	33.4–56.0	50 (14.8)	13.4 (10.3–16.6)

ALM: appendicular lean mass; GS: gait speed; HGS: handgrip strength; ALM/height^2^: appendicular lean mass/height^2^; ALM/BMI: appendicular lean mass/body mass index; TUG: timed up-and-go; ROC: receiver operating characteristics. Missing data: HGS *n* = 1 man; TUG *n* = 3 women, 2 men; GS *n* = 323 women, 7 men.

**Table 4:** Agreement between sarcopenia prevalence estimates according to different international and population-specific cut-points. Data are presented as κ, 95% confidence intervals and *p*-values.

Women	ALM/height^2^_EWGSOP1_ + HGS	ALM/height^2^ _EWGSOP2_ + HGS	ALM/BMI _FNIH_ + HGS	ALM/height^2^ + HGS	ALM/height^2^ + TUG	ALM/BMI + HGS		
ALM/height^2^ + HGS	0.2 (0.0–0.5)	0.4 (0.0–0.7)	0.1 (−0.1–0.2)	-				
*p-value*	<0.001	<0.001	0.02					
ALM/height^2^ + TUG	0.3 (0.0–0.5)	0.2 (0.0–0.4)	0.1 (0.1–0.2)	0.4 (0–0.7)	-			
*p-value*	<0.001	0.001	0.32	<0.001				
ALM/BMI + HGS	0.2 (0.0–0.4)	0.2 (0.0–0.4)	1	0.1 (0.0–0.3)	0.1 (−0.1–0.2)	-		
*p-value*	0.001	0.001	<0.001	0.02	0.3			
ALM/BMI_ROC_ + HGS	0.3 (0.1–0.5)	0.3 (0.1–0.5)	0.7 (0.5–0.8)	0.1 (0.0–0.3)	0.1 (0.0–0.2)	0.7 (0.5–0.8)		
*p-value*	<0.001	<0.001	<0.001	<0.001	0.11	<0.001		
**Men**	**ALM/height^2^_EWGSOP1_ + HGS**	**ALM/height^2^_EWGSOP1_** **+ GS**	**ALM/height^2^_EWGSOP2_ + HGS**	**ALM/BMI _FNIH_** **+ HGS**	**ALM/height^2^** **+ HGS**	**ALM/height^2^ + TUG**	**ALM/heigh^2^ + GS**	**ALM/BMI** **+ HGS**
ALM/height^2^ + HGS	0.8 (0.6–1.0)	0.4 (0.2–0.7)	0.4 (0.1–0.7)	0.1 (-0.1–0.3)	-			
*p-value*	<0.001	<0.001	<0.001	0.13				
ALM/height^2^ + TUG	0.4 (0.2–0.7)	0.5 (0.4–0.7)	0.3 (0.0–0.6)	0.1 (−0.1–0.2)	0.5 (0.3–0.8)	-		
*p-value*	<0.001	<0.001	<0.001	0.26	<0.001			
ALM/height^2^ + GS	0.5 (0.3–0.8)	0.7 (0.4–0.9)	0.5 (0.1–0.8)	0.1 (−0.1–0.3)	0.7 (0.4–0.9)	0.8 (0.6–1.0)	-	
*p-value*	<0.001	<0.001	<0.001	0.11	<0.001	<0.001		
ALM/BMI + HGS	0.2 (0.0–0.3)	0.1 (0.0–0.3)	0.02 (0.0–0.1)	0.3 (0.1–0.4)	0.1 (0.0–0.3)	0.0, (−0.8–0.2)	0.07 (0–0.2)	-
*p-value*	<0.001	0.02	0.30	<0.001	0.001	0.39	0.09	
ALM/BMI_ROC_ + HGS	0.2 (0.1–0.3)	0.2 (0.0–0.3)	0.0 (0.0–0.1)	0.2 (0.1–0.3)	0.1 (0.0–0.1)	0.0 (0–0.1)	0.1 (0.0–0.2)	0.7 (0.6–0.8)
*p-value*	<0.001	0.001	0.04	<0.001	0.172	0.182	0.02	<0.001

EWGSOP: European Working Group on Sarcopenia in Older People; FNIH: Foundation for the National Institutes of Health; ALM: appendicular lean mass; GS: gait speed; HGS: handgrip strength; ALM/height^2^: appendicular lean mass/height^2^; ALM/BMI: appendicular lean mass/body mass index; TUG: Timed-up-and-go. For population-specific cut-points, low values corresponded to T-score < −2, except where indicated as ROC (derived from receiver operating characteristic curves).

## Data Availability

The data presented in this study are available on request from the corresponding author.

## Abbreviations

ALM	Appendicular lean mass
ANZSSFR	Australian and New Zealand Society for Sarcopenia and Frailty Research
BMI	Body mass index
CI	Confidence interval
DXA	Dual-energy X-ray absorptiometry
EWGSOP	European Working Group on Sarcopenia in Older People
FNIH	Foundation for the National Institutes of Health
GOS	Geelong Osteoporosis Study
GS	Gait speed
HGS	Handgrip strength
TUG	Timed up-and-go
ROC	Receiver operating characteristic
